# Potential acceleration performance of a 256‐channel whole‐brain receive array at 7 T

**DOI:** 10.1002/mrm.27519

**Published:** 2018-09-26

**Authors:** Arjan D. Hendriks, Peter R. Luijten, Dennis W. J. Klomp, Natalia Petridou

**Affiliations:** ^1^ Department of Radiology, Imaging Division University Medical Center Utrecht Utrecht Netherlands

**Keywords:** 2D CAIPIRINHA, 7 T, 256‐channel receive coil, massive receive, parallel imaging, phased array

## Abstract

**Purpose:**

Assess the potential gain in acceleration performance of a 256‐channel versus 32‐channel receive coil array at 7 T in combination with a 2D CAIPIRINHA sequence for 3D data sets.

**Methods:**

A 256‐channel receive setup was simulated by placing 2 small 16‐channel high‐density receive arrays at 2 × 8 different locations on the head of healthy participants. Multiple consecutive measurements were performed and coil sensitivity maps were combined to form a complete 256‐channel data set. This setup was compared with a standard 32‐channel head coil, in terms of SNR, noise correlation, and acceleration performance (g‐factor).

**Results:**

In the periphery of the brain, the receive SNR was on average a factor 1.5 higher (ranging up to a factor 2.7 higher) than the 32‐channel coil; in the center of the brain the SNR was comparable or lower, depending on the size of the region of interest, with a factor 1.0 on average (ranging from 0.7 up to a factor of 1.6). The average noise correlation between coil elements was 3% for the 256‐channel coil, and 5% for the 32‐channel coil. At acceptable g‐factors (< 2), the achievable acceleration factor using SENSE and 2D CAIPIRINHA was 24 and 28, respectively, versus 9 and 12 for the 32‐channel coil.

**Conclusion:**

The receive performance of the simulated 256 channel array was better than the 32‐channel reference. Combined with 2D CAIPIRINHA, a peak acceleration factor of 28 was assessed, showing great potential for high‐density receive arrays.

## 1 INTRODUCTION

The concept of using arrays of multiple receive‐coil elements to enhance SNR has already been around for a couple of decades.^1^ Receive arrays can greatly enhance SNR in brain imaging, as compared with the use of a standard quadrature head coil covering the same area.^2^ The benefits of using up to 32 receive elements for brain imaging have been demonstrated extensively.^3^, ^4^, ^5^, ^6^, ^7^, ^8^ However, just a few brain imaging studies have explored the advantages of receive coils with more than 32 elements.^9^, ^10^

When combined with parallel imaging, the improved SNR from receive coil arrays can be traded for a faster acquisition time, enhancing the temporal resolution, while maintaining high spatial resolution. When examining dynamic and spatially detailed brain functions using functional MRI (fMRI), the combination of both a high temporal resolution and a high spatial resolution is essential. High‐density receiver arrays allow for high acceleration factors with reduced g‐factors and very high resolutions.^11^, ^12^ However, fMRI with both a high temporal resolution (< 1 second) and a high spatial resolution (< 1 mm) combined, is rarely seen, despite the latest developments in the field of parallel imaging and modern imaging setups.

From theory, the benefit of increasing the number of receive elements is two‐fold. First, a gain in SNR can be achieved.^2^ Second, it enables improved encoding acceleration performance,^13^ as can be exploited by using parallel imaging techniques.

The number of receive elements can be increased and the coil size can be reduced accordingly, as long as the individual coils are in tissue load dominance and coil resistance losses are minimal.^14^, ^15^ This would result in an improved SNR close to the coils, with hardly losing sensitivity further away from the coils.^10^, ^16^ If the individual coils are made too small, (electronic) coil resistance loss dominates the sample noise,^17^ which would degrade the total SNR. This relation determines that there is an optimal number of coils at which the achievable SNR is maximized. Different 3T studies use receive arrays with high numbers of receive elements, sometimes also referred to as massively parallel MRI detection,^18^ and show a clear benefit in using 64 receive channels,^9^, ^19^ 96 channels,^10^ and even 128 channels.^20^, ^21^

The theoretical maximum achievable SNR limit, described as the ultimate SNR, is investigated by previous studies.^22^, ^23^ These studies simulate a spherical object at varying field strengths, sphere sizes, and acceleration factors. Among other results, it was shown that a 32‐channel receive setup at 3 T already closely approaches the ultimate SNR in the center of the phantom, but is still far away from the ultimate SNR at the regions near the surface of the phantom. Additionally, at higher field strengths, such as 7 T, the difference between the SNR acquired with 32 channels and the ultimate SNR increases, and more coils would be required to approach the ultimate SNR at both the surface of the phantom and at intermediate distance from the center.

In contrast to increasing the number of receive elements, present‐day efforts in the MRI community are geared towards parallel imaging techniques.^24^, ^25^, ^26^ These techniques can greatly reduce the total acquisition time, pushing the limits of temporal and spatial resolution. Parallel imaging techniques reduce acquisition time by uniformly undersampling k‐space data acquired with receive arrays, while maintaining the maximum k‐values to keep the full spatial resolution. Early and widely implemented parallel imaging techniques are SENSE,^27^ GRAPPA,^28^ and the more recent CAIPIRINHA (controlled aliasing in parallel imaging results in higher acceleration).^29^ When using the undersampling patterns of 2D CAIPIRINHA,^30^ aliasing patterns can be controlled, posing less restriction on coil sensitivity variations. Specifically, the noise amplification due to receiver coil configuration and geometry (g‐factor) can be reduced.

Ultrahigh field strengths have the commonly acknowledged advantage of an intrinsic SNR gain and an increase in BOLD sensitivity. In addition, at ultrahigh field strength the RF eddy current interaction with human tissue enhances the spatial variance of B_1_^‐^ fields,^31^, ^32^ paving the way to higher acceleration factors as well.^23^ This interaction also increases the tissue load dominance of the receive coils, meaning that smaller coil elements can be used without contributing substantially to the noise (i.e., low noise figure).^33^ At 7 T, when positioned close to the human head, coil elements can be as small as 2 cm^2^, with a noise contribution of less than 20%.^12^ Considering the surface area of the head, the number of coil elements for full‐brain MRI could potentially be increased from 96 at 3 T to about 256 at 7 T without contributing substantial extra noise. Moreover, realization of such a 256‐channel array comes closer with the recent developments around the implementation of a digital receive pipeline for 7T scanners.^34^ Despite these technical advances and theoretical predictions, it still has to be demonstrated that higher numbers of small receive elements can contribute substantially to acceleration for whole‐brain imaging at 7 T.

The aim of this study is to investigate the possible acceleration performance of a 256‐channel receive coil array at 7 T in combination with the parallel imaging methods SENSE and 2D CAIPIRINHA, for 3D data sets. Measurements were performed with 2 × 16 channel arrays, which were shifted over the head to simulate a 256‐channel receive coil array. As a reference, the achieved sensitivity and acceleration performance is compared with a 32‐channel head coil at 7 T, for both parallel imaging methods. In line with observations of SENSE‐optimized RF coils,^35^, ^36^ we opted for a receive array design with gaps between the circumferentially distributed coil elements. Alternatively, overlapped designs could show higher nonaccelerated SNR at the periphery, although at the cost of increased g‐factors at high acceleration. A gapped design was used to improve SENSE performance, as this study focused on maximizing the acquisition acceleration at maintained SNR rather than increasing intrinsic SNR without acceleration.

## 2 | METHODS

### 2.1 | Overview

A 256‐channel receive array was simulated by shifting 2 small 16‐channel high‐density receive arrays to different locations on the head of healthy participants. On the same day a measurement with a standard Nova 32‐channel receive coil setup was performed, which was taken as a reference. To check reproducibility, the measurement session was repeated with a second participant for both setups. Before scanning, the participants gave written informed consent, as required by the ethical committee of the University Medical Center Utrecht. Coil sensitivity reference scans and noise prescans were used to obtain the data. In postprocessing steps, the SNR maps, noise correlations, and g‐factor maps were calculated for both coil setups (simulated 256‐channel setup, standard head coil setup) and for the 2 parallel imaging techniques (2D SENSE, 2D CAIPIRINHA).

### 2.2 | Setup

**Figure 1:**
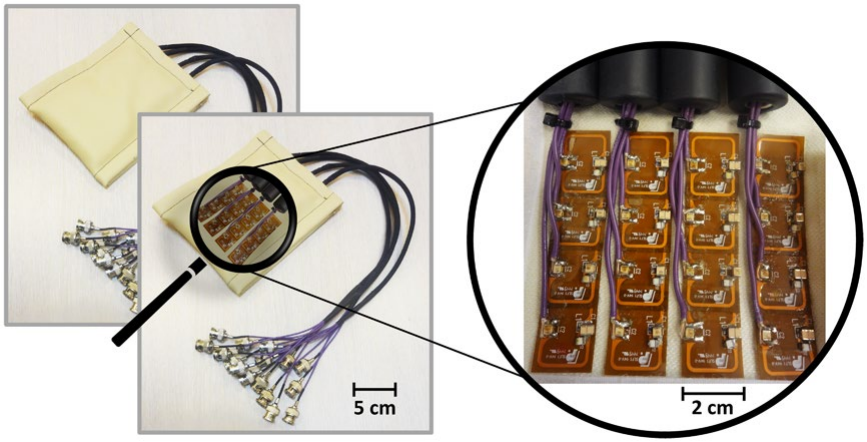
Two high‐density receive arrays with 16 channels each (left) and a zoomed image of the coil elements (right). These coil arrays can be used for high spatial resolution and high temporal resolution functional MRI imaging. However, with just 16 channels, the brain coverage of the arrays is limited. In this study, high‐density receive arrays are used to simulate a 256‐channel coil with full brain coverage by shifting the coils to different locations on the head as illustrated in Figure 2

A 7T Achieva MRI scanner (Philips Healthcare, Best, Netherlands) was used for data acquisition in combination with a volume transmit/receive coil (Nova Medical, Wilmington MA, USA), driven by two 4‐kW amplifiers. The volume transmit/receive coil provided whole brain excitation. This system was consecutively equipped with the following 2 receive‐only setups. The first receive setup consisted of 2 high‐density 16‐channel receive arrays (MR Coils BV, Zaltbommel, Netherlands). The receive coil array consisted of small, 1.5 × 2 cm^2^, elements arranged in 4 flexible modules of 4 elements each (Figure 1), which were overlapped (0.5 cm). The elements were decoupled from one another by high impedance preamplifier decoupling. Each module had an outer dimension of 2 × 8 cm^2^ and was flexible so that it could be positioned within 1 mm of the head. The use of passive detuning circuitry and fuses was avoided, because this would degrade the unloaded Q factor of the coil too much. For safety, active real‐time PIN diode control surveillance was added, to immediately stop the scanner when the diode control demonstrated a malfunction. Previous publications^12^, ^37^, ^38^ describe and evaluate the high‐density receive arrays in more detail. For completeness we also checked the unloaded and loaded Q‐values of the coil element.

The second receive setup consisted of a standard 32‐channel receive head coil (Nova Medical, Wilmington MA, USA). This coil array was used as the reference for comparison with the simulated 256‐channel array. The 32‐channel coil array consisted of large square loops of approximately 5 × 4 cm^2^. For decoupling purposes the coil elements are overlapping by 1 cm in the feet–head direction. The coil elements are arranged in 10 modules of 2 to 4 loops. Additional details of this receive coil were published previously.^39^ Both receive setups were connected to the same preamplifiers and receive pipeline, to make the noise figures of the preamplifiers comparable.

### 2.3 | Measurement procedure

Two participants were scanned. For each participant, both the 2 × 16 channel receive array and the Nova 32‐channel head coil setup were used consecutively. For both participants, the measurement procedure involved 4 steps.

**Figure 2:**
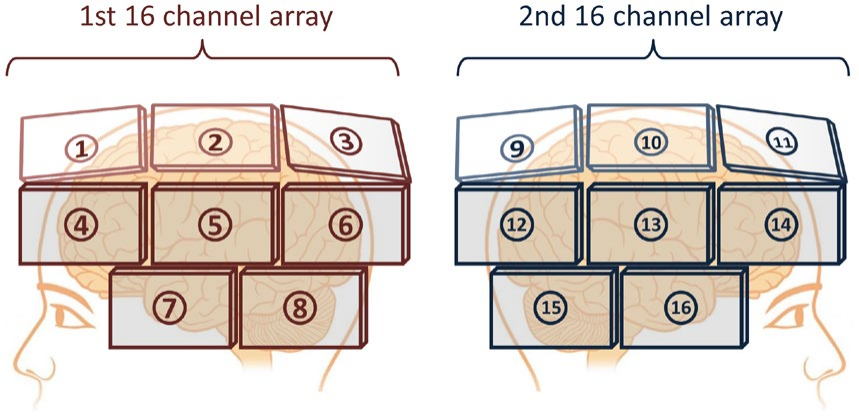
A schematic showing how two 16‐channel coil arrays were shifted to 16 different positions on the head, contributing to the 256‐channel array simulation. To acquire all 256 sensitivity maps, only 8 reference scans (8 shifts of 32 channels) were needed. To acquire all cross terms in the noise correlation matrix, 120 noise scans (120 shifts of 32 channels) were required

First, an initial anatomical scan was acquired to position the imaging volume (3D gradient echo, TE/TR = 2.2/4.8 ms, voxel size = 1.5 × 1.5 × 1.5 mm^3^, FOV = 25 × 25 × 20 cm^3^, total acquisition time = 2 minutes). Second, to assess the performance of a 256‐channel whole‐brain receive array, measurements were performed with two 16‐channel receive arrays, which were positioned 8 times to 16 different locations on the head of the participant (Figure 2). For each location, a coil sensitivity reference scan (details below) with noise prescan was acquired, yielding a total of 8 reference scans, each containing signal from 32 channels, contributing to the formation of a virtual 256‐channel coil.

Third, the two 16‐channel receive arrays were shifted to every other possible combination of 2 arrays on 16 head positions $\left(\begin{array}{c} 16\\ 2 \end{array}\right)$. For each combination, a short noise scan was performed. In total, 120 noise scans were required. The noise scan was a Philips prescan containing 20 000 noise points for each channel, acquired with a 400‐kHz sampling rate. These noise points and the noise points of the 8 reference scans were used for the determination of the noise covariance and noise correlation between channels.^40^

Finally, a measurement with the second receive setup was performed, which uses the standard 32‐channel receive head coil. As this setup was connected to the same receive pipeline, the behavior of the preamplifiers and receiver gain settings was comparable. For this setup, a single reference scan with noise prescan was acquired for the same participant. In all scans, an automated power optimization was performed to assure that flip angles remained constant over the different setups. The transmit coil loading was comparable for the different receive setups. No substantial changes in the transmit power were observed, as changes in transmit power were within 5% range.

### 2.4 | Coil sensitivity reference scans

Coil sensitivity reference scans^27^ were acquired by successive signal reception with the volume transmit/receive coil and either the high‐density surface receive arrays or the standard head coil. Data from the reference scans contained information about the coil sensitivity for each coil element individually and a “background” image acquired by the volume transmit/receive coil. Each reference scan contained a preceding noise scan. The reference scans were acquired with a 3D gradient‐echo sequence with the following parameters: TE/TR = 1.22/8.0 ms, voxel size = 2 × 2 × 2 mm^3^, FOV = 20 × 20 × 20 cm^3^, and total acquisition time = 1 minute.

### 2.5 | Postprocessing and simulations

After scanning, coil sensitivity maps were constructed from the coil sensitivity reference scans. The sensitivity maps were calculated using ReconFrame (GyroTools LLC, Zürich, Switzerland) by dividing the individual complex coil images by the complex image acquired with the volume coil.^27^

The resulting 8 sets of 32 (2 × 16)‐channel sensitivity maps were combined to form a simulated 256‐channel receive array. To correct for potential head displacement between the reference scans, images acquired for each coil position were aligned to the images of the first position using the alignment modules of the AFNI software package.^41^ Alignment parameters (rotation and translation) were calculated using the reconstructed whole‐brain magnitude images from the reference scan. These alignment parameters were first applied to the whole‐brain complex images (to visually inspect the alignment quality) and then to the sensitivity matrices per coil position. The resulting 256 aligned coil sensitivity maps and individual coil images of the head formed the basis of the virtual head coil. The same procedure was applied to construct the sensitivity maps of the standard 32‐channel head coil, with the exception that a coil alignment correction was not required in this case, as the signal from these 32 channels was already acquired simultaneously.

To analyze the data, SNR maps were calculated from the aligned individual complex coil images for both receive setups. The individual complex coil images were combined using SNR‐weighted channel addition, as described by the following equation:

$$SNR=\frac{\sum_{n=1}^{N_{c}}\left|w_{n}I_{n}\right|}{\sqrt{\sum_{n=1}^{N_{c}}\left(w_{n}^{2}{\sigma_{n}}^{2}\right)}}$$

where *I_n_* defines the set of complex images of the *n*th individual coil element, *w_n_* are the weight factors that are spatially varying per voxel and per coil element, *N_c_* represents the total number of coil elements, and *σ_n_* is equal to the standard deviation over the selected noise region for each coil element. The selected noise region consisted of more than 40 000 points (18 × 18 × 128) for each coil element. The noise region was placed in the corner of each individual coil element image in image domain and care was taken to avoid placing the noise region over ghosting and filter artefacts. The SNR difference of the simulated 256‐channel array compared with the 32‐channel head coil was calculated by first aligning the 2 data sets and then dividing the same slices of both data sets. With the exception of noise correlation, the SNR maps could be compared directly, as the 2 receive setups were used together with the same transmit coil and were consecutively connected to the same receive pipeline (same receiver gain and preamplifiers). Three brain regions of interest (ROIs) were analyzed by segmenting the brain in a periphery ROI, a center ROI and a midpoint ROI, based on the anatomy of the reconstructed magnitude images of the reference scan. The 95% range and average gain factor in SNR, excluding noise correlation, was calculated for both regions. Additionally, axial SNR profiles through the center of the brain were computed for both receive setups.

The noise covariance and noise correlation matrix were calculated from 120 separate noise scans and the 8 noise prescans included in the reference scans. The 8 noise prescans from the reference scan were used to fill the 8 submatrices of 32 channels on the diagonal of the correlation matrix. The 120 separate noise scans were used to fill the remaining cross terms of the matrix. Because the noise scans were acquired consecutively, the noise coefficients were first calculated per scan individually, only correlating simultaneously acquired noise. Afterward, the calculated noise coefficients of these separate scans were combined to form 1 matrix of 256 × 256 channels.

The g‐factor was obtained from the sensitivity maps according to Pruessmann et al.^27^ The noise covariance matrix was included in the g‐factor calculations, as a noise prewhitening step. Because each individual sensitivity map was noise‐correlated with only 32 simultaneously acquired channels, only the inner eight 32‐channel submatrices on the diagonal of the noise covariance matrix were required to prewhiten the sensitivity maps for the g‐factor calculations. To simulate the SENSE and CAIPIRINHA parallel imaging techniques, 2 directions were undersampled. These directions were anterior–posterior (AP) and feet–head (FH), as these directions contained the most coil elements. The range of the simulated undersampling factors (R) was 1 to 10 in the AP direction and 1‐6 in the FH direction. Multiple g‐factor calculations were performed for different combinations of 2D SENSE acceleration factors and different 2D CAIPIRINHA undersampling patterns. The reconstruction pipeline for 2D SENSE and 2D CAIPIRINHA undersampled datasets was similar between both techniques, as in essence only the indexing of the aliased voxels had to be adjusted.

## 3 | RESULTS

**Figure 3:**
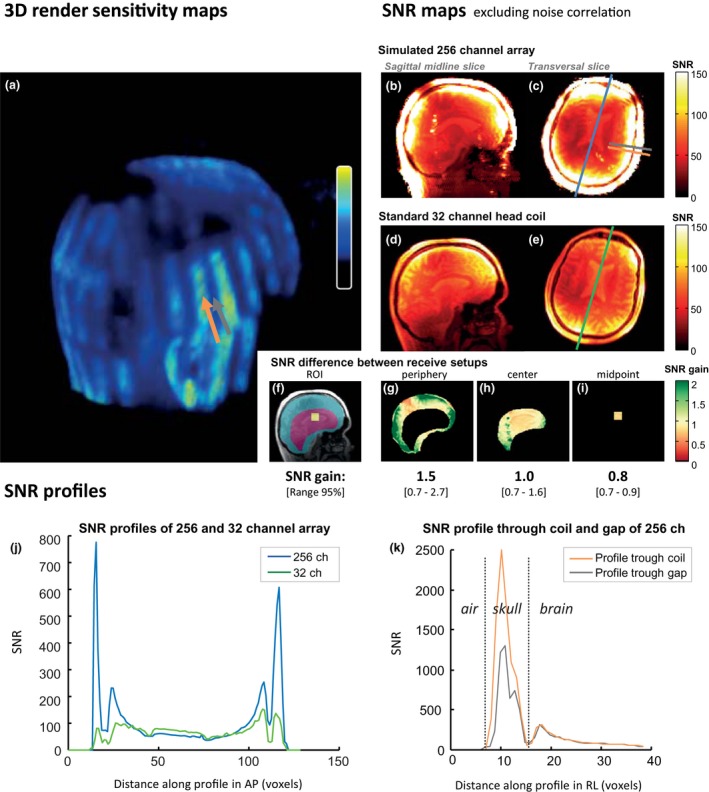
Overview of the simulated 256‐channel coil. The 3D render of combined sensitivity maps (a) was thresholded to display only the high sensitivity values, making the 256‐coil elements visually observable as high‐intensity stripes over the head. The SNR of both the simulated 256‐channel array and the standard 32‐channel head coil is evaluated by comparing the SNR maps (b‐e), SNR difference in 3 regions of interest (ROIs) (f‐i), and SNR profiles (j). All of the SNR maps, SNR‐difference ROIs, and the SNR profiles show a pattern of a high SNR gain for the simulated 256‐channel array in the periphery, a comparable SNR over a large part of the center of the brain, and a reduction in SNR in the worst‐case midpoint of the brain. Additionally, SNR profiles (k) were drawn through a coil element (orange line) and through a gap (gray line) of the simulated 256‐channel array. These profiles show that the nonuniformity in sensitivity caused by the coil gaps does not go much further than the skull. The locations of the selected profiles are also indicated in (a) and (c). AP, anterior–posterior; RL, right–left

An impression of the coil distribution of the 256‐coil elements over the head can be seen in the surface‐rendered sum of the sensitivity maps (Figure 3a). The figure shows an intensity‐scaled 3D rendering of the acquired MR data. The 256 (8 × 32)‐coil array elements, with a loaded and unloaded Q of 89 and 248, respectively, fit the head of the participant and columns of overlapping elements can be clearly distinguished as lines over the head. An evaluation of the performance of the simulated 256‐channel receive coil array can be found in the cross sections of the SNR maps (Figure 3b‐e). A sagittal and axial slice through the midsection of the brain are depicted, providing a representative overview of the SNR for the full 3D data set, including the lower values in the center of the brain. The SNR maps of both the simulated 256‐channel receive array and the Nova 32‐channel coil are displayed. Because both receive coil setups were attached to the same receive pipeline and only 1 transmit setup was used, the SNR maps can be compared between setups. However, it should be noted that noise correlation could only be partially incorporated for the simulated 256‐channel coil. In comparison with the standard head coil, especially at the periphery of the brain, the SNR for the simulated 256‐channel coil is higher, which is seen for both of the illustrated slices. The SNR difference between the simulated 256‐channel coil and the standard 32‐channel coil is visualized by dividing the SNR maps of both setups. Center and periphery ROIs (Figure 3f) are drawn in the midsection slice to quantify the average and range of the SNR differences between setups (Figure 3g‐i). In the brain periphery (Figure 3g), when partially neglecting the effects of noise correlation, the SNR difference for the simulated 256‐channel coil as compared with the standard 32‐channel coil is a factor of 1.5 on average, ranging from 0.7 (absence of coils) to 2.7 (close to coils). Moreover, directly next to the coils, on the brain surface, higher gains in SNR are observed. In the center of the brain, when selecting a large central ROI (Figure 3h), the SNR difference is a factor 1.0 on average, ranging from 0.7 to 1.6 with an average of 1.0. In the worst‐case midpoint of the brain (Figure 3i), there is a reduction of SNR, with a SNR difference of 0.8 on average, ranging from 0.7 to 0.9. The SNR profiles (Figure 3j) show the same trend. High SNR values are found for the simulated 256‐channel array in the skull and periphery of the brain, whereas the SNR is slightly lower in the middle of the brain when comparing both receive setups. For the 256‐channel array, the SNR profiles that go through a gap between coil elements have similar SNR inside the brain, as profiles that go through a coil element itself (Figure 3k). The influence of the gaps between the coil elements on SNR remains superficial and does not reach much further than the skull.

**Figure 4:**
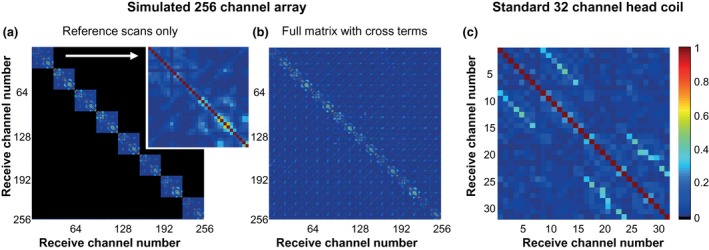
Noise correlation matrix of the simulated 256‐channel array is displayed, as acquired from the reference scans only (a) and when including the extra 120 noise scans (b). For comparison, the correlation matrix of the standard 32‐channel head coil (c) is also displayed. For visual inspection, a zoomed image of channels 1 to 32 of the 256‐channel array is shown in the inset of (a). The noise correlation matrix of the simulated 256‐channel receive array was filled in blocks of 32 channels consecutively (and not simultaneously). A total of 120 noise scans, obtained using different coil positions on the head, were required to measure all cross terms. When comparing both setups, the average noise correlation of the off‐diagonal elements is similar for the 256‐channel coil as compared with the 32‐channel coil

The measured noise correlation matrix (Figure 4) is obtained from the 8 reference scans and the 120 subsequent noise scans acquired with the two 16‐channel surface arrays. The noise correlation coefficients of the 8 reference scans are placed in 8 submatrices of 32 channels on the diagonal (Figure 4a). The 120 noise scans were used to further fill all the cross‐terms (Figure 4b). The overall noise correlation is low and similar across the elements. To illustrate this, the average magnitude and maximum magnitude of the noise correlation coefficients was calculated. For the simulated 256‐channel array, the average of the off‐diagonal noise correlation coefficients is 0.027 (2.7%, -31.5 dB). When only incorporating the noise coefficients that were measured together with the sensitivity maps (inner 8 submatrices of 32 channels), the average correlation is 0.065 (6.5%, -23.7 dB).

For the standard 32‐channel head coil the average noise correlation is 0.054 (5.4%, -25 dB). The maximum noise correlation coefficient is 0.67 for the simulated 256‐channel coil and 0.42 for the standard 32‐channel head coil.

**Figure 5:**
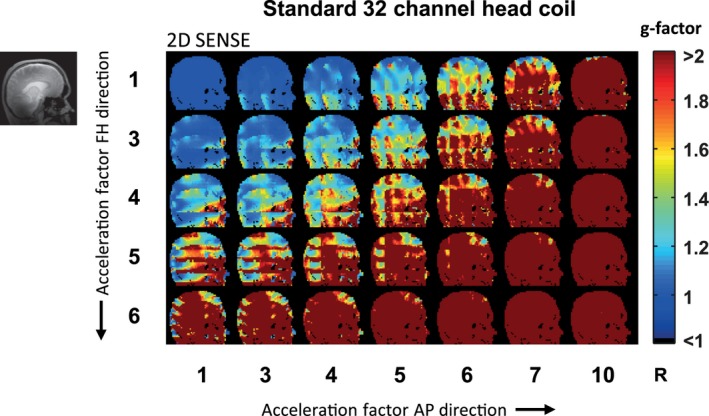
g‐factor maps for the standard 32‐channel head coil using the undersampling patterns of 2D SENSE. Additionally, the gray scale image (left) shows the corresponding anatomical position of the selected slice located close to the midsection of the brain. The colored images show the g‐factor maps, which are thresholded at a factor of 2 (color bar). The acceleration factor increases in both feet–head direction (rows), and anterior–posterior direction (columns). The maximum achievable acceleration factor for the standard 32‐channel head coil, using SENSE and g‐factors smaller than 2, is 9 (SENSE 3 × 3)

**Figure 6:**
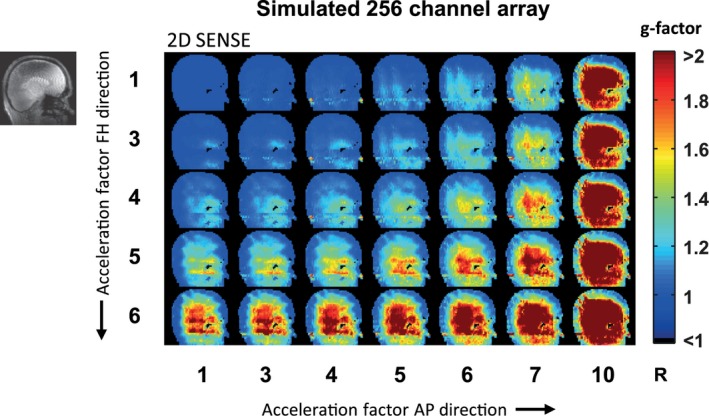
g‐factor maps for the simulated 256‐channel receive array using the undersampling patterns of 2D SENSE. The maximum achievable acceleration factor for the simulated 256‐channel receive array, using SENSE and g‐factors smaller than 2, is 24 (SENSE 4 × 6). This is substantially higher than the maximum SENSE acceleration of the standard 32‐channel head coil of 9 (SENSE 3 × 3) displayed in Figure 5

**Figure 7:**
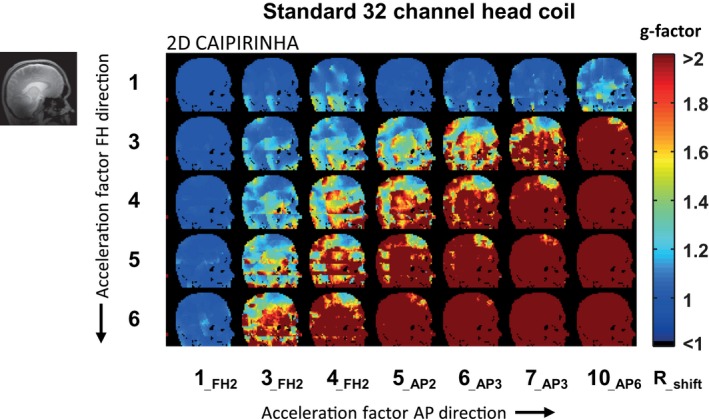
g‐factor maps for the standard head coil using the undersampling patterns of 2D CAIPIRINHA. Specific patterns can be referred to by R^total^ (undersampling method R^FH^ x R^AP^__CAIPIshift_). The maximum achievable acceleration factor for the standard 32‐channel head coil, using CAIPIRINHA and g‐factors smaller than 2, is 12 (CAIPIRINHA 4 × 3__FH2_). Note that this is higher than the maximum acceleration factor achieved with SENSE using the same setup

**Figure 8:**
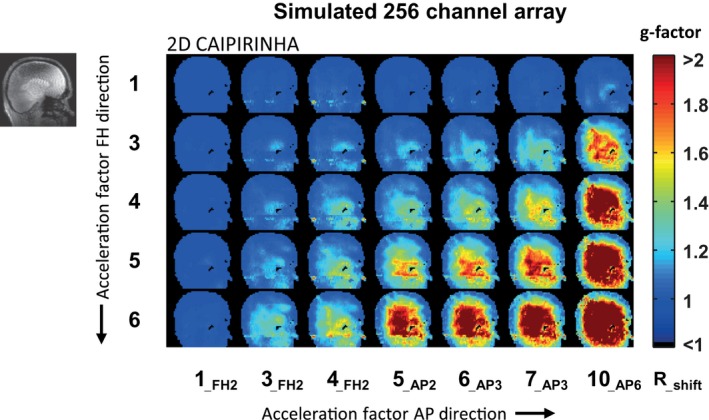
g‐factor maps for the simulated 256‐channel receive array using the undersampling patterns of 2D CAIPIRINHA. The maximum achievable acceleration factor for the simulated 256‐channel receive array, using CAIPIRINHA and g‐factors smaller than 2, is 28 (CAIPIRINHA 4 × 7__AP3_)

The calculated g‐factor maps when using SENSE undersampling patterns are shown for different acceleration factors, for both the standard 32‐channel head coil (Figure 5) and the simulated 256‐channel coil (Figure 6). The calculated g‐factor maps when using 2D CAIPIRINHA undersampling patterns are shown as well (Figures 7 and 8). Different acceleration factors are displayed in the FH and AP direction. At low or no acceleration, the g‐factor is similar for both setups. Overall, it can be seen that the g‐factor, corresponding to noise amplification, increases with the acceleration factor. For both setups, the g‐factor penalty diminishes when the total acceleration factor is distributed over 2 directions instead of 1, by using either 2D SENSE (Figures 5 and 6) or 2D CAIPIRINHA (Figures 7 and 8). The increase in g‐factor is spatially confined to the center of the brain for the simulated 256‐channel coil, while more diffuse across the brain for the 32‐channel head coil. The use of 2D CAIPIRINHA has a lower g‐factor penalty than the use of SENSE, which is seen for both setups. When considering a g‐factor of less than 2 (to avoid excessive noise amplification), the maximum achievable acceleration for the standard 32‐channel head coil is 9 (SENSE 3 × 3) and 12 (CAIPIRINHA 4 × 3__FH2_). For the simulated 256‐channel head coil this is 24 (SENSE 4 × 6) and 28 (CAIPIRINHA 4 × 7__AP3_). Note that even at very high accelerations (> 28), the g‐factor at the periphery of the brain is still below 2 for the 256‐channel coil. As a side note, the SENSE and CAIPIRINHA maps are identical for the first 3 g‐factor maps of the top row, where the shift direction is not undersampled (CAIPI_dir_: FH; R_FH_: 1), as no k‐space shift is performed in a direction without undersampling. The results are shown for a sagittal slice through the midsection of the brain, representative of the “worst‐case” situation. Slices that are positioned further away from the center have either an equal or better g‐factor performance. Results are illustrated for 1 participant; similar results were obtained for the second participant.

## 4 | DISCUSSION

The aim of this study was to assess the potential gain in acceleration performance of a 256‐channel high‐density receive coil array at 7 T, combined with the parallel imaging methods 2D SENSE and 2D CAIPIRINHA for 3D data sets. To realize this, measurements were performed with two 16‐channel gapped receive arrays consisting of small coil elements, which were used as basic building blocks to simulate a 256‐channel head coil. The g‐factor maps indicate that the 256‐channel head coil array can deliver more than a 2‐fold improvement in acceleration performance, as compared with the standard 32‐channel head coil. In addition, for both the standard head coil and the simulated 256‐channel array, a 2D CAIPIRINHA sampling pattern can significantly improve the SNR or acceleration performance. Overall, a remarkable maximum acceleration factor of 28 is estimated. This suggests that whole‐brain high‐density receive arrays combined with fast parallel imaging acquisition methods show great potential for fMRI with high spatial and high temporal resolution.

### 4.1 | Signal‐to‐noise ratio

The SNR of the simulated 256‐channel array is high, especially at the periphery of the brain, as can be observed in the SNR maps. However, in these SNR maps not all noise correlation terms could be included, because the reference scans were obtained consecutively. In the periphery of the brain, the average gain in SNR of the simulated 256‐channel array is a factor of 1.5 and goes up to a factor of 2.7 in tissue close to the coil elements. Up to a certain depth, measured from the coil elements, smaller‐sized coil elements gain SNR, which can also be seen in the SNR profiles. However, for very small coils, this substantial gain will be restricted to few centimeters away from the coil. In this study, the worst‐case midpoint region of the brain shows an SNR reduction by a factor of 0.8 on average (ranging from 0.7 to 0.9), whereas the SNR of a larger central region of the brain is comparable between setups with a factor of 1.0 on average (ranging from 0.7 to 1.6). The SNR in the worst‐case midpoint region is lower as compared with the standard 32‐channel head coil, raising questions about the optimum 7T head coil configuration, coil element size, and the number of channels to achieve maximum intrinsic SNR. The noise figure of the simulated 256‐channel coil is expected to increase by 20%, due to the smaller element size,^12^ which would be evident especially in the center of the brain. The relatively good SNR values in the larger central region of the brain might be explained by the fact that the 256‐channel surface coil elements are closer to the head than the 32‐channel elements. Coil proximity improves the coil loading, enhancing the SNR.^42^ If the array was designed for maximum intrinsic SNR excluding acceleration, then our coil element size may be too small. However, the motivation of our study was to maximize acceleration performance while maintaining SNR.

### 4.2 | Noise correlation

To obtain optimal SNR, it is necessary to ensure that the noise from channel to channel is largely uncorrelated.^43^ In this study, the noise correlation between individual coils is low, with average noise correlation coefficients of 0.027 and 0.054 for the 256‐channel array and the 32‐channel head coil, respectively.

### 4.3 | Acceleration performance

The acceleration performance was assessed based on the g‐factor maps. For both 2D SENSE and 2D CAIPIRINHA acquisition schemes, acceleration factors for the simulated 256 channel array are 2‐fold higher than the standard 32‐channel head coil, considering a maximum acceptable g‐factor of 2. An estimated peak acceleration factor of 28 was found when using 2D CAIPIRINHA. The use of 2D CAIPIRINHA is desirable over 2D SENSE, because it has lower g‐factors due to more optimal distribution of aliasing patterns. For the simulated 256‐channel array, especially at the periphery of the brain, the g‐factor is well below 2. This is in accordance with the high receive sensitivity at that location. Even at very high accelerations such as 42, the g‐factor at the edge of the brain is still below 2 for the 256‐channel coil. For studies that are only interested in the periphery of the brain, as is the case for some cortical fMRI studies, this would indicate that potentially even higher accelerations than a factor of 28 are possible.

### 4.4 | Comparison with state‐of‐the‐art high‐density receive arrays

Previous work at 3 T of Wiggins et al. shows that a 96‐channel receive array for the head improves imaging performance, in comparison to an identically sized 32‐channel coil.^10^ When following this direction at 7 T, smaller coil elements can be used without contributing substantially to the noise. Below, a comparison of the receive performance of the simulated 256‐channel array to the 96‐channel array is given in terms of SNR, average noise correlation, and acceleration performance. For the simulated 256‐channel array, an average SNR increase of a factor 1.5 is seen in the periphery of the brain as compared with the 32‐channel coil. For 96‐channel array at 3 T, an SNR increase of a factor of 1.4 was reported, as compared with an equally sized 32 channel coil. The average off‐diagonal noise correlation coefficient is low for the 256‐channel coil, with a value of 0.027. For the 96‐channel coil this is 0.148. The maximum SENSE acceleration performance was evaluated by comparing the g‐factor maps with a g‐factor of less than 2. A maximum acceleration factor of 24 (SENSE 4 × 6) can be achieved for the simulated 256‐channel array, as measured in a volunteer. For the 96‐channel coil a maximum acceleration factor between 9 (SENSE 3 × 3) and 16 (SENSE 4 × 4) was reported, as measured in a brain phantom.

A possible explanation for the large increase in acceleration factor for the 256‐channel array (as compared with the 32‐channel coil and also as compared with previous work) could be the coil array design. First, a high number of coil elements was used here, enabling the acquisition of more data with receivers that have different spatial sensitivities, which can be translated to higher attainable acceleration factors. Second, no overlap between coil elements was used in the transverse direction. The gap between elements not only benefits SENSE performance,^35^ but also facilitates flexibility of the coil array, hence assuring a tighter fit to the head. Third, using preamplifier decoupling and cable management of the 2 × 16 receive channels, RF coupling between adjacent coil elements was aimed to be low. The cables of the 2 × 16 channel receive array are guided in sets of 4 channels per cable trap (Figure 1), reducing the cable coupling burden. A future step in cable management is the implementation of a digital receive pipeline, which would allow data from multiple receive channels through fiber optic cables, drastically reducing the amount of RF cables further.

### 4.5 | Methodological considerations

From a methodological point of view, the 8 reference scans that formed the basis of the simulated 256‐channel coil were acquired consecutively, excluding most noise correlation terms in the g‐factor calculations. Nevertheless, coil coupling is expected to be highest between neighboring coils, and this was taken into account in this study by partially filling the noise correlation matrix (diagonally) with the noise coupling measured from the 2 × 16 channel sets (Figure 4a). Still, coupling between directly neighboring coils from adjacent measurement sets were excluded from the simulation. Additionally, noise correlation may even increase if incorrect cable management is provided. The coils from different measurement sets are at a further distance physically, so it should be possible to maintain negligible noise coupling between these elements with correct cable management (e.g., when digital receivers are placed before the cables get in close proximity). Considering the impact of noise correlation on image SNR in general, one needs to consider true coil sensitivities, including noise correlations. However, as described by others, in general there is almost no difference in SNR from omitting the coil‐noise matrix from the reconstruction algorithm for coils that are well decoupled.^44^

The transmit power drive scale did not change more than 5% between receive setups, which excludes a large bias in the intrinsic SNR comparison caused by changes in B_1_ field. However, once a full 256‐channel array would be in place, the B_1_ level of transmit coil can be affected significantly. The sum of all small potential couplings of the transmit coil to copper, cables, and cable traps of a full receiver setup may require more RF power for the same B_1_ field. To mitigate this, stronger RF amplifiers might be necessary.

The maximum acceleration factor in this study was assessed by evaluating the g‐factor maps. In practice, the acceleration factor and SNR are not solely dependent on the g‐factor. When the k‐space signal is undersampled during acquisition, there is also an inherent SNR loss (√R) from acquiring fewer samples. The image SNR (SNR_0_) decreases with the g‐factor and the acceleration factor (g·√R). However, the time‐course SNR (tSNR), which is important for fMRI applications, decreases less rapidly.^45^ Depending on the application and imaging settings, an optimal acceleration factor can be chosen. Generally, g‐factor maps are a good indication of the potential acceleration performance of a coil.

### 4.6 | Choices for coil element design and array configuration

In this study a number of design choices were made including coil element size, the number of coil elements, and the amount of overlap between elements. All choices were made to target high acceleration factors. The large number of small coil elements used in this study made it possible to achieve a high acceleration factor of 28. Despite these high acceleration factors achieved, it can be questioned what the most optimal design and array configuration is for a field strength of 7 T. In addition, an optimal coil design to achieve maximum acceleration with parallel imaging may be different than the optimal coil design for maximum intrinsic SNR without acceleration.^40^ There is still room for improvement in the design and shape of individual coil elements, as these were not optimized in length versus diameter. Furthermore, overlapped designs^9^, ^10^ could have a different optimal number and size of coil elements at 7 T as compared with SENSE‐specific gapped designs.^35^, ^36^ More advanced coil arrangements and designs, which for example use a combination of different coil sizes and overlap, may outperform our proposed setup in the future. This could lead to other, potentially even higher acceleration factors, as demonstrated with single‐echo acquisition techniques.^19^

### 4.7 | Future and technical considerations of 256 channels

From a practical point of view, a number of engineering challenges may emerge with respect to the construction of a 256‐channel coil. First, small coil elements are required, so they can cover the head in a quantity of 256. In this study, small 1.5 × 2 cm^2^‐sized elements were used that can be fitted tightly over the head.

Second, the receive pipeline connecting the 256‐channel array should also be physically able to connect and subsequently preprocess the signal from all of these channels. A digital receive pipeline seems essential, because it enables information from multiple receive channels to be transported through 1 fiber optic cable. The design goal is to convert the signal from analog to digital as early in the receive pipeline as possible, minimizing the amount of RF cables and their length, therefore reducing the coupling between cables. Preamplifier boards can be designed to be small enough to be stacked tightly in the scanner bore behind the head coil, diminishing the excessive use of cables.

Third, a large number of independent receiver channels can also have practical limitations in terms of handling and processing a substantially larger amount of independent data streams. In particular, reconstruction speed can decrease considerably. At the same time, however, digital receive and computer hardware are evolving to be able to handle these kinds of data flow. Furthermore, data‐size reduction techniques, such as array compression,^46^ have proven to be very effective in reducing data size even before image reconstruction.

## 5 | CONCLUSIONS

Overall, the results showed superior receive performance of the simulated 256‐channel receive array on acceleration performance. This holds not only in comparison to the standard 32‐channel head coil, but also as compared with other reported high‐density receivers in literature. The results in this study suggest that the benefits achieved in spatial and temporal fMRI resolution by using the high‐density 32‐channel configuration can be extended to 256 channels with full head coverage. In conclusion, the simulated 256‐channel head coil shows great acceleration possibilities. Together with 2D CAIPIRINHA, a peak acceleration factor of 28 can be achieved, revealing high potential for whole‐brain high‐density receive arrays combined with fast parallel imaging acquisition methods to measure brain function at high spatial and high temporal detail.
